# Impact of the TEAM Wheels eHealth manual wheelchair training program: Study protocol for a randomized controlled trial

**DOI:** 10.1371/journal.pone.0258509

**Published:** 2021-10-13

**Authors:** Ed Giesbrecht, Julie Faieta, Krista Best, François Routhier, William C. Miller, Maude Laberge

**Affiliations:** 1 Department of Occupational Therapy, University of Manitoba, Winnipeg, Manitoba, Canada; 2 Department of Health and Rehabilitation Sciences, University of Pittsburgh, Pittsburgh, Pennsylvania, United States of America; 3 Department of Rehabilitation, Université Laval, Quebec City, Quebec, Canada; 4 Centre interdisciplinaire de recherche en réadaptation et en intégration sociale (CIRRIS), Centre intégré universitaire de santé et de services sociaux de la Capitale-Nationale (CIUSSS-CN), Quebec City, Quebec, Canada; 5 Department of Occupational Science & Occupational Therapy, University of British Columbia, Vancouver, British Columbia, Canada; 6 Département d’opérations et systèmes de décision, Université Laval, Quebec City, Quebec, Canada; Universitat de Valencia, SPAIN

## Abstract

**Background:**

Variable, and typically inadequate, delivery of skills training following manual wheelchair (MWC) provision has a detrimental impact on user mobility and participation. Traditional in-person delivery of training by rehabilitation therapists has diminished due to cost, travel time, and most recently social distancing restrictions due to COVID-19. Effective alternative training approaches include eHealth home training applications and interactive peer-led training using experienced and proficient MWC users. An innovative TEAM Wheels program integrates app-based self-training and teleconference peer-led training using a computer tablet platform.

**Objective:**

This protocol outlines implementation and evaluation of the TEAM Wheels training program in a randomized control trial using a wait-list control group.

**Setting:**

The study will be implemented in a community setting in three Canadian cities.

**Participants:**

Individuals ≥ 18 years of age within one year of transitioning to use of a MWC.

**Intervention:**

Using a computer tablet, participants engage in three peer-led teleconference training sessions and 75–150 minutes of weekly practice using a video-based training application over 4 weeks. Peer trainers individualize the participants’ training plans and monitor their tablet-based training activity online. Control group participants also receive the intervention following a 1-month wait-list period and data collection.

**Measurements:**

Outcomes assessing participation; skill capacity and performance; self-efficacy; mobility; and quality of life will be measured at baseline and post-treatment, and at 6-month follow-up for the treatment group.

**Impact statement:**

We anticipate that TEAM Wheels will be successfully carried out at all sites and participants will demonstrate statistically significant improvement in the outcome measures compared with the control group.

## Introduction

Use of a manual wheelchair (MWC) to address mobility impairment is both common and growing. Close to 200,000 community-dwelling Canadians use a MWC, and the number of wheelchair users has risen by 30% over the previous ten years [1]. Despite increasing prevalence of MWC provision, users often experience restricted social participation and mobility because they are not provided with the *skills* to independently, safely, and effectively use their MWC [2]. Low self-efficacy, or a lack of *belief* in one’s ability to use a wheelchair, can further compromise participation [3].

Among all people with a disability, wheelchair users are more likely to experience home accessibility issues, require assistance with more activities of daily living on a more frequent basis, and have greater out-of-pocket expenses to obtain assistance [4]. In Canada, over 95% of MWC users required caregiver assistance with at least one major life activity including 37% for basic mobility [4]. Restricted mobility is associated with reduced participation and social connectedness [5], leading to isolation, stress and low self-esteem impacting quality of life [6]. One group adjusting to MWC use (stroke survivors) also identified substantial restriction in *caregivers’* social roles and increased burden of care [7]; these are typically family members or unpaid individuals. In Canada, the annual incidence of tips or falls is 5.2%, with 4.2% resulting in injury and 2.5% requiring an ER visit [8]. In the United States, wheelchair-related accidents result in 1 death per week [9].

To address these troubling outcomes, a key component of best-practice service provision is individualized, user-centered skills training following MWC procurement [10]. The Wheelchair Skills Program (WSP) is an example of a comprehensive, structured and evidence-informed resource for skills training content and delivery [11]. Two systematic reviews of structured MWC skills training programs found them to be safe, practical and effective [12,13]. Research studies with the WSP report statistically significant and clinically meaningful improvement in skill capacity during inpatient rehabilitation [14,15] and in the community [16,17]. However, training is typically delivered by a physical or occupational therapist and requires 4 to 8 sessions, with at least 10–12 hours of training recommended to ensure safe and proficient performance [18]. In hospital, when most MWCs are prescribed, competing discharge demands are often prioritized over training. A survey of 68 Canadian rehabilitation centres reported over half of therapists spent two hours or less on MWC training and 18% provided no skills training [19]. Funding for community-based service is in decline and insufficient to support therapist-intensive training either before or after discharge [20,21]. Time and travel demands for MWC user and therapists make traditional training cost-prohibitive [22]. Long wait lists and inaccessibility of rehabilitation services, particularly in rural areas, exacerbates the problem [22,23].

Alternative and disruptive rehabilitation approaches are required that are clinically effective, cost-effective, and sustainable. Two strategies have demonstrated potential to address these barriers and support equitable care for new MWC users: delivery in the community via eHealth and use of peer trainers. A tablet-based MWC skills home training program, administered and monitored by an occupational therapist, was shown to be feasible and effective, and perceived as highly beneficial among middle and older aged MWC users [4]. In a randomized controlled trial, statistically significant improvements in participation, wheelchair self-efficacy and skill safety were reported, with large effect sizes [24]. A peer-led wheelchair skills training program is also feasible to administer [25] and has demonstrated statistically significant improvements in wheelchair skills capacity, performance and self-efficacy in a randomized control trial [26]. Two influential enablers of self-efficacy, *vicarious experience* (the observation of comparable peers achieving success) and *verbal persuasion* (the encouragement and confidence of others), are identified in Bandura’s Social Cognitive Theory [27]. We intentionally leveraged this effect through the use of a peer trainer, who has experiential knowledge and similar characteristics as the target population [28], and can cultivate social connections through sharing of realized knowledge.

These two training approaches are compatible and synergistic, providing an opportunity to ameliorate training issues among MWC users in a cost-effective way delivered at the most appropriate time and in the most appropriate context. *Training to Enhance Adaptation and Management for Wheelchair users* (TEAM Wheels) was designed as an enhanced version of the earlier eHealth (computer tablet) home training application [29] and integrated as part of a peer training intervention [26]. The TEAM Wheels program was originally to be delivered using 3 in-person sessions with the peer trainer, consistent with both pilot studies, as well as in-person data collection. Prior to implementation, the COVID-19 worldwide pandemic created conditions that undermined our ability to conduct such in-person activities. In addition, the need for social distancing and heightened safety considerations further highlighted why MWC users might avoid clinic settings. These circumstances prompted a rapid shift in delivery; however, these changes were also consistent with long-term goals of adapting TEAM Wheels for rural and remote delivery. This adapted approach replaced in-person peer training sessions with tablet-based teleconferencing.

**Fig 1:**
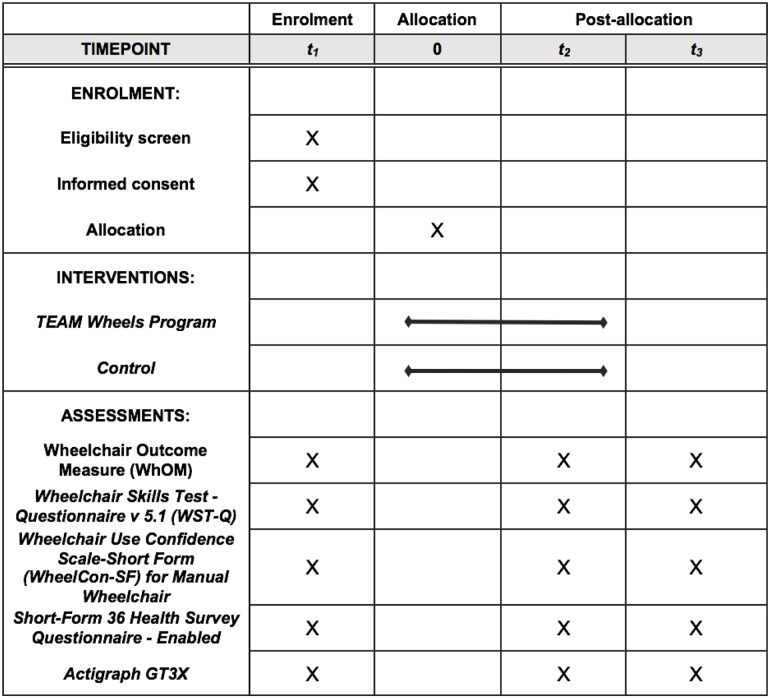
SPIRIT schedule of enrolment, interventions, and assessments.

While both peer-led and tablet app-based MWC skills training have been assessed [30,31], the combine value of these approaches is yet to be reported. Our purpose is to evaluate the impact of a 1-month, peer-led eHealth training program (TEAM Wheels) on satisfaction with activity participation and related rehabilitation outcomes among individuals transitioning to MWC use, compared with current wheelchair training practice. This manuscript describes the protocol that will be used to evaluate this impact in a randomized control trial (Fig 1).

### Study objectives

#### Primary objective

We hypothesize that at post-treatment, the primary outcome of wheelchair-related participation will be significantly higher in the TEAM Wheels group and that post-treatment improvements will be retained at 6-month follow up.

#### Secondary objective

We also hypothesize that the TEAM Wheels program will demonstrate clinically and statistically significant differences at post-treatment on secondary outcome measures of wheelchair-specific self-efficacy; skill capacity and performance; health-related quality of life; and objective measurement of mobility. In addition, we hypothesize that post-treatment improvements will be retained at 6-month follow up.

## Materials and methods

### Design

**Fig 2:**
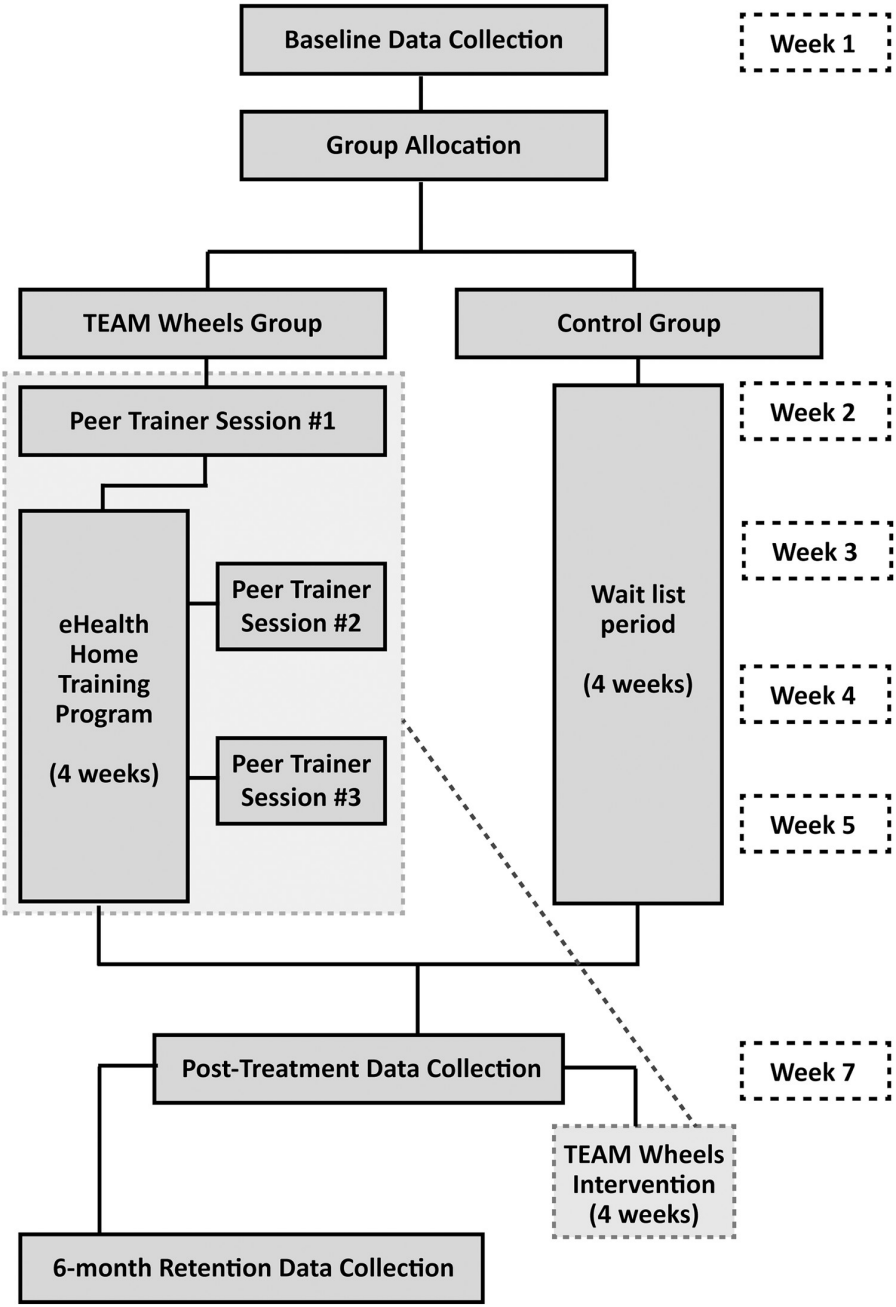
CONSORT flow diagram.

This multisite randomized controlled trial will compare a 4-week MWC training intervention and wait-list control group among individuals transitioning to MWC use. Consent, data collection and intervention components will all be conducted without in-person interaction following national health recommendations during the COVID pandemic [32]. Baseline data (T1) will be collected and entered into a secure database, and participants allocated to their group assignment. Control group participants are placed on waitlist while treatment group participants engage in a series of 3 teleconference training sessions (roughly 10 days apart) and a home training application (all conducted using a computer tablet) over a 4-week period. Following a 1-week consolidation window, post-intervention data collection (T2) is conducted. The control group participants will receive the intervention following T2 collection to ensure equity. Follow-up data collection (T3) will be conducted with the treatment group participants after 6 months (Fig 2). All study personnel involved in collecting data will remain blinded to group allocation.

### Participants and recruitment

Participants will meet the following inclusion criteria: 18 years and older, ≤1 year since obtaining their first MWC; able to propel with both arms; and residing in the community. Exclusion criteria include: a health condition that might interfere with training; insufficient cognitive or language abilities (as determined by capacity to respond to the screening questions); or concurrently receiving supplementary MWC training.

Recruitment will follow a convenience sampling technique across all study sites (Quebec, Winnipeg, and Vancouver). Active recruitment strategies that engage health care providers, rather than relying on potential participants to initiate contact, are highly effective [33,34], therefore, a number of tailored recruitment strategies will be employed. Using established relationships with health care facilities, allied health professionals at each site will approach patients who meet the eligibility criteria to explain the TEAM Wheels program and obtain consent for investigator contact. A recruitment brochure will be mailed out to individuals who, in the past year, have received a MWC through a provincial wheelchair provider program. Additional recruitment strategies include advertisements posted in public venues; advocacy and consumer groups; wheelchair vendors; outpatient therapy service providers; and social media pages using multimedia advertisements (e.g., infographics, short animations, and personnel videos).

Participants will be provided with a copy of the *Information and Consent Form* via post or email. A research assistant will review the form via telephone or secure (PHIA/FIPPA-compliant) videoconferencing and participants will confirm consent via their preference of post, email, audio recording or secure online format. Once enrolled, participants will be randomized to either the waitlist control or intervention group using a 1:1 allocation ratio between groups. A central computerized randomization process will be designed by a statistician, with an undisclosed block size and stratified by site.

The combined results of our previous feasibility studies found a large effect size (*f* = 0.47) for the WhOM and correlation between measures (*R*^2^) was 0.63. Using a comparable effect size (*f* = 0.45) and measure correlation (0.6), α = 0.05, and 90% power for a between-factors ANOVA repeated measures design, a sample size of 42 is required (G*Power v.3.1.9.2). Adjusting for 20% loss to attrition, a total sample size of 54 was determined (i.e., 27 per intervention and control group). We anticipate recruiting 1–2 participants/per site/per month, estimating 12 months required to recruit the sample. Incorporating the treatment and 6-month follow-up periods, the maximum data collection period should be 19 months. While we have built in a 20% attrition rate, our feasibility studies reported 5–10% drop-out rates [24,25] and, with the provision of the TEAM Wheels program to control group participants after data collection, we anticipate limited attrition.

### Intervention

#### Intervention group

At least 2 peers will be hired at each site, offering multiple trainer attributes (i.e., male/female; younger/older). Participants will pre-select a peer trainer based on a biosketch to optimize training effect; comparability in age has been identified as preferential among older adults [35] and influential to self-efficacy [27]. Participants will receive a package that includes a Samsung Tab A™ 10.1” computer tablet and instructional material prior to meeting with their peer trainer. Participants who do not have home Wi-Fi will also receive a mobile hotspot device to provide Internet access. Peer trainers will conduct 3 real-time video conferences 60–90 minutes in duration using the Microsoft Teams™ platform on the computer tablet provided; these sessions will occur approximately 10 days apart. The peer role is to collaborate with participants to identify personal goals; tailor the training program to these goals; orient the participant to the eHealth training program; monitor home training activity; and provide education and training on safety, skill performance and strategies to address both accessibility and environmental barriers. Session 1 will focus on goal setting, safe training practices, and orientation to the eHealth home training application.

The tablet is preloaded with a custom-built Android software program that includes over 200 videos components (available in both French and English). A rigid platform and tablet holder mounts on the participant’s lap using an adjustable strap, with the tablet secured using hook and loop fasteners. The application content is organized into nine folders addressing safety, skills, and maintenance; examples of content are provided in Table 1. Videos include instruction, peer demonstration, and practice activities and games. The application provides feedback on weekly and total training activities as well as virtual awards for completing training activities. Participants are instructed to practice with the training application for 75 to 150 minutes per week. Based upon motor learning principals [36], training sessions of 15–30 minutes 1–2 times per day, 3–5 days per week will be encouraged. Participants’ training activity on the tablet will regularly upload to a secure server; the peer trainer monitors this activity online and may adapt the program or follow-up with the participant based on their progress data. Participants and peer trainers can also exchange direct messages during the training using the chat function on Microsoft Teams™. Opening the application triggers a query about how many minutes the participant has spent practicing without the tablet; whether they have experienced a tip or fall since their last training session; and if so, whether this was related to practicing a new skill.

**Table 1:** Examples of skills included in home training app.

Folder	Example Skills Included
1. Safety	Equipment; Supervision/Spotting; Tips & Falls; Training Strategies
2. Basic Components	Wheel locks; Drive wheels; Casters; Body position & weight shift
3. Skills 1	Hand position; Propulsion techniques; Coasting
4. Skills 2	Propelling backwards; Making turns; Moving sideways; Reaching
5. Skills 3	Avoiding obstacles; Popping casters; Crossing gaps; Soft surfaces
6. Skills 4	Shallow inclines; Cross slopes; Slippery surfaces; Doors
7. Skills 5	Drag turns; Steep inclines; Wheelies
8. Skills 6	High obstacles; Steps and stairs
9. Maintenance	Weekly, monthly and annual maintenance activities

#### Control group

The control group will not receive any study-related intervention during the four-week period between baseline and post-treatment (alternative MWC training interventions are an exclusion criterion and so will not impact control participants). At present, there is little MWC training provided following the initial prescription of a MWC to a new user [37]. Therefore, this 4-week “waiting” period is reflective of current practice. Following completion of the study (i.e., after post-treatment data collection), control group participants will receive the TEAM Wheels training program intervention. The waitlist control design was selected as an equitable approach based on the positive outcomes reported in our previous studies [24,31].

Safety issues relevant to TEAM Wheels do not exceed the risks that MWC users typically encounter on a daily basis. However, participant safety will be addressed in several ways. The initial eHealth training module addresses safe wheelchair use and guidelines for safe practice; this module must be completed before access to the remaining content is released. Participants will be encouraged to identify a family member, friend, or caregiver who would be involved in video conference and home training sessions to provide safe “spotting”. Participants will receive a spotter strap (i.e., device to prevent rearward tips) and protective propulsion gloves. The initial video conference will integrate safe wheelchair operation, use of a spotter strap and safe spotting technique. Should unsafe practices be observed during video conferences, the peer-trainer will provide immediate corrective feedback. The peer-trainer will report any adverse events (e.g., tip/fall) arising from the tablet queries or conversation with the participant to the study team. Severe adverse events will be reported to the data safety and monitoring board and the ethics review board.

### Data collection

Sociodemographic characteristics will be collected at T1 (1 week prior to the intervention). Five outcome measures (available in both French and English) will be administered at T1, T2 (1-week post-intervention), and (for treatment group participants) at T3 (6-month post-treatment). Due to COVID-related restrictions, a research assistant (RA) blinded to allocation will coordinate questionnaire administration via telephone or secure video conferencing. Participants will be provided with the questionnaires in advance (via post or email) and have the option to complete in pen/paper, online using a secure Qualtrics™ platform, or collaboratively with the RA by telephone or secure video conference. The RA will input the data using Qualtrics™ or confirm completeness and follow-up, if necessary, for participants who chose to complete questionnaires online.

### Outcome measures

The sociodemographic questionnaire includes age, sex, gender, marital status, highest level of education, primary diagnosis related to MWC use, and length of time using the MWC. Measurement properties of the remaining measures are summarized in Table 2.

**Table 2:** Measurement properties of outcome measures.

Outcome	Measurement Tool	Attributes
Satisfaction with Participation	Wheelchair Outcome Measure (WhOM) [38–41]	Strong reliability (Test-retest ICC = 0.83–0.88; Inter-rater ICC = 0.90–0.91) [39] Strong validity (correlations with LIFE-H: *r*_*s*_ = 0.3–0.5) [39] (correlation with QUEST *r*_*s*_ = 0.36–0.45) [42] Clinically relevant change score of 1.6 [39]
MWC Skills	Wheelchair Skills, Test—Questionnaire v 5.1 (WST-Q) [43]	Strongest psychometric properties of MWC skills outcomes in 2 systematic reviews [44,45] Strong reliability (test-retest ICC = 0.90; intra-rater ICC = 0.96; and inter-rater ICC = 0.97 administration) [43] Correlates highly with WST objective testing version (*r* = 0.89) and imposes lower administration time/burden than the objective Wheelchair Skills Test [46]
MWC Self-Efficacy	Wheelchair Use Confidence Scale-Short Form (WheelCon-SF) for Manual Wheelchair [47]	More responsive than full 65-item version [47] Test-retest reliability (ICC = 0.98) and internal consistency (Cronbach’s alpha = 0.95) [48]
Health Related Quality of Life	Short-Form 36 Health Survey Questionnaire [49,50]	Assesses perceived satisfaction with both physical and emotional health [49,50] Good internal consistency measured within a Spinal Cord Injury population (Cronbach’s alpha 0.72~0.98) [51]
Objective MWC Mobility	Actigraph GT3X	Correlates with mobility and community engagement [52] Valid and reliable measurement of MWC movement [53,54] Measure wheelchair movement with high accuracy (90–96%) across a variety of activities and contexts [54] Can calculate “bout” frequency (meaningful transitions between functional activities) [53]

Note: MWC = manual wheelchair.

#### Primary outcome: *Satisfaction with participation*

The International Classification of Functioning, Disability and Health (ICF) identifies participation as “involvement in a life situation,” [55] including the subjective experience and performance of important activities and roles. The Wheelchair Outcome Measure (WhOM) is a patient-oriented measure of satisfaction with participation using a wheelchair, specifically related to relevant activities inside and outside of the home. Using a semi-structured interview format, respondents identify 10 activities (5 performed inside and 5 performed outside of the home), rating their satisfaction with performance on an 11-point scale (0–10) for each activity.

#### Secondary outcomes

The *Wheelchair Skills Test—Questionnaire* v 5.1 (WST-Q) asks respondents to evaluate their ability to perform 32 discrete items (skills) related to negotiating their environment in a MWC. In accordance with the ICF, there are two scales: respondents rate their *Capacity* (i.e., what they can do) as well as *Performance* (i.e., how frequently they do it). Each item is rated on a scale from 0–2 and total skill Capacity (0–100%) and Performance (0–100%) scores are calculated.

Self-efficacy, or belief in one’s ability to use a wheelchair [56], is a significant determinant of participation frequency and mediator of skill capacity [57]. The *Wheelchair Use Confidence Scale-Short Form (WheelCon-SF)* is a self-report questionnaire with 21 statements related to confidence using a wheelchair in various activities and environments. Items are rated on a scale from 0 (“not confident”) to 10 (“completely confident”), providing a total mean score (0–10) [47].

Health utility measurement is useful when evaluating the impact of rehabilitation interventions. National guidelines for healthcare economic analyses strongly advocate the use of a validated measure of health-related quality of life (HRQL), which can be converted to quality-adjusted life years (QALY) gained to fully inform funding decisions [58]. The Short-Form 36 Health Survey Questionnaire is a measure of health status across the following domains: physical function, physical limitations, emotional limitations, pain, social function, energy, perceived health, and mental health [49,50]. The literature identifies inherent wording bias of the mobility-related SF-36 test items, which presume ambulation as a norm [59,60]. Consequently, we opted to use the *Enabled* version that explicitly acknowledges use of a mobility aid and replaces the words *climbing* and *walking* with *going* [61].

To triangulate measurement of improvement in wheelchair skill performance and participation outcomes, accelerometry data (Actigraph™, Pensacola FL) will be used to *objectively measure MWC mobility* [52]. An Actigraph™ datalogger is attached to the MWC drive wheel, passively collecting data without impeding MWC operation. A second datalogger is worn on the user’s arm; retrospective data comparison between devices will delineate wheelchair movement generated exclusively via self-propulsion. Algorithms are used to convert data into total and mean values of distance, speed, and “bouts” of activity (i.e., meaningful transitions between functional activities) [53]—parameters that reflect mobility patterns and activity of MWC users which we expect to change as a result of improvements in skill and participation.

### Statistical analyses

Sociodemographic information will be summarized with descriptive statistics. After testing for assumptions and outliers, between-groups (intervention vs control) comparison of change over time will be conducted using Generalized Linear Modeling (GLM) for participation; MWC skill capacity and performance; self-efficacy; health-related quality of life; and objective MWC mobility parameters (α = 0.05) [62]. Variables previously identified in the literature as relevant covariates (i.e., age and function as measured by baseline WST-C) will be controlled for. Model fit (*R*^2^), regression coefficients (CI_95%_), and effect sizes (*F*^2^) will be assessed with Maximum Likelihood methods. Data will initially be disaggregated by sex and gender; if analyses prove non-significant, the data will be reaggregated. A repeated-measures ANOVA will be used to evaluate retention of outcomes at follow-up for treatment group participants. Additional post-hoc sub-analyses will investigate the impact of site (using 2-way ANOVA) and length of MWC use covariate (using ANCOVA), although the study is powered only for the primary analysis. Descriptive data will report on recruitment by sex, relative to prevalence data for MWC use [1] to inform generalizability of findings and health services equity balance [63].

### Trial status

Peer trainers are being recruited and will be trained between January and April 2021. We anticipate initiating recruitment and data collection in March 2021.

## Discussion

The TEAM Wheels program provides a novel approach to community based MWC skills training. This program could be implemented as a strategy for continuum of rehabilitation care as individuals’ transition to community reintegration. The use of eHealth technology enables delivery in a home setting and could potentially mediate rehabilitation challenges experienced by those living in rural and remote communities. The COVID-19 pandemic and need for social distancing expedited our transition from a hybrid model (i.e., in-person peer training + eHealth home training app) to a comprehensive eHealth delivery of both peer and home training components.

TEAM Wheels is designed to address several gaps in current MWC service provision. Gaps addressed include lack of access to MWC training options during a period of widespread health concerns and social distancing, a paucity of individualized intervention designs, and the need for a more holistic approach to education on advanced MWC skills training. The anticipated benefits of the TEAM Wheels program for novice MWC users includes greater satisfaction with participation; improved wheelchair skills performance and self-efficacy; increased mobility; and enhanced quality of life. In addition, the TEAM Wheels program is expected to have positive, ancillary benefits for MWC users who provide the peer-training component of the program. Specifically, peer trainers have additional opportunity to attain employment and to engage in social activity whilst observing stringent safety precautions. In sum, the TEAM Wheels training program is designed as a viable solution to the distinct educational needs of wheeled mobility device users in a pandemic world.

### Peer training approach

The use of a peer-training approach adds to the effectiveness, feasibility, and sustainability of the TEAM Wheels program. Social isolation is reported to yield negative health outcomes to vulnerable populations [64]. It is therefore important to consider how health and quality of life interventions might continue to incorporate meaningful social interaction in a way that adheres to safety precautions. Peer-training provides this social interaction, and further, pairs new MWC users with trainers whom they are likely to identify with, enhancing their own self-efficacy.

Occupational and physical therapists are typically involved in wheelchair service delivery. However, a recent study of rehabilitation centres across Canada indicated few therapists receive specialized MWC training beyond their professional entry-to-practice education. In contrast, peer-trainers have accrued valuable experience related to navigating their community with a MWC and becoming proficient with MWC skills. With training, peers can be uniquely qualified to teach MWC skills and to convey their knowledge to novice MWC users. A health care equation can consider outcomes of healthcare as the dividend and the cost of care over time as the divisor [65,66]. Considering this formula, an effective healthcare delivery method (peer-trainers) that is cost-effective (reducing the need for clinician trainers) could yield improved value in the care provided to novice MWC users.

### Community-based intervention

The development of a MWC skills training program that integrates training within the home context of the user is expected to be highly effective in preparing MWC users to navigate their community. Beyond the current pandemic-related social distancing considerations, more than 6.3 million Canadians reside in rural regions [67]. Geography is an important consideration for MWC service provision; TEAM Wheels participants do not incur the expense and inconvenience of attending to a clinic or rehabilitation center can leverage the benefits and training safety in an authentic home environment.

### Sustainability

A strength of TEAM Wheels is its focus on sustainability. The TEAM Wheels program integrates two effective training approaches that we anticipated to be highly sustainable: a consumer grade, off-the-shelf technology platform (i.e., tablet) and existing community based MWC experts who can effectively deliver, and benefit from, the program. The home training software application can be upgraded and can be customized in the future to address a variety of needs such as propulsion strategies (e.g., hemiplegic and foot propellers) and populations (e.g., pediatrics; individuals with amputation or stroke). While TEAM Wheels currently leverages the visual and physical accessibility that a large tablet offers, it has the potential to be delivered using a variety of mobile device platforms that are already familiar to many users. As mobile device technology updates and upgrades over time, the TEAM Wheels software can be refined to be compatible with future operating systems and screen parameters.

Peer-trainers are an essential element of the TEAM Wheels program. The experience of being a peer mentor can be rewarding; peer mentors report feeling valued for their ability to create hope and share their lived experience with others [68]. We expect that peer-trainers in the TEAM Wheels program will have similar positive experiences and see this as important and meaningful vocation. Trainees within the TEAM Wheels program may, over time, become proficient with MWC skills to the extent that they have the opportunity to assume a peer trainer position, organically propagating the reach of the TEAM Wheels program.

### Limitations

Potential limitations include challenges in recruitment within and across sites. We will be utilizing existing relationships with hospital systems and rehabilitation organizations as well as social media platforms to optimize recruitment. Use of an eHealth platform has the potential for technology related issues (system freezing, update requirements, lost or broken tablets). To address this, we have updated content of the training application and will have replacement tablets on hand. In addition, training materials have been developed to support peer trainers and participants to utilize the technology platforms involved.

## Conclusions

The TEAM Wheels program was designed to provide accessible and sustainable community-based wheelchair skills training to novice MWC users. This eHealth program combines a peer-trainer teleconference and app-based home training component delivered via a computer tablet. The effectiveness of the TEAM Wheels program will be evaluated using a randomized control trial carried out in three Canadian provinces. Participation in TEAM Wheels is expected to yield statistically significant and clinically meaningful improvements in participation, skill capacity, self-efficacy, mobility and quality of life.

## Supporting information

S1 Checklist SPIRIT 2013 checklist: Recommended items to address in a clinical trial protocol and related documents. (DOC)

S1 File (PDF)
