# Supplementary material for: Impact of the TEAM Wheels eHealth manual wheelchair training program: Study protocol for a randomized controlled trial
Source: PLoS One. 2021 Oct 13;16(10):e0258509. doi: 10.1371/journal.pone.0258509 (PMC8513836; doi:10.1371/journal.pone.0258509)
Supplement: S1 File — (PDF) [file pone.0258509.s002.pdf]

## Overview

Many Canadians receive a manual wheelchair (MWC) to address mobility impairment, yet they experience restricted social participation and mobility because they are not provided with the *skills* to independently, safely, and effectively use their MWC. Low self-efficacy, or a lack of *belief* in one's ability to use a MWC, can further compromise participation. Suboptimal use of a MWC results in substantial social costs such as reduced engagement in meaningful activity, social isolation, higher caregiver burden, and a poor use of financial resources considering the cost of MWC acquisition and attendant care. Access to comprehensive *wheelchair training* is constrained by the expense and limited availability of skilled therapists; demands of patient and/or clinician travel; lack of training opportunities when returning to the community. In addition, current Government of Canada, Public Health Agency of Canada (PHAC), and provincial health authority restrictions and guidelines related to the ongoing coronavirus (SARS-CoV-2) pandemic, including physical distancing and promotion of non-contact services, have compromised in-person wheelchair service delivery, in part because this is a vulnerable population with underlying medical conditions that are at increased the risk of developing severe complications from COVID-19. *Training to Enhance Adaptation and Management for Wheelchair users* (TEAM Wheels) is a 4-week, community-based, tailored *e*Health program integrating peer-led and independent home training components to optimize learning while limiting the time demands of health care professionals and mitigating the risk of COVID-19 for participants, peer-trainers, and research staff. The *e*Health program is delivered using a 10" computer tablet with a custom home-training application and a secure teleconferencing application. The tablet mounts conveniently on the wheelchair user's lap for "in-chair" training, enabling MWC users to learn and practice mobility skills in authentic contexts of home and community. The interactive home-training application includes a structured curriculum with over 200 video clips featuring education, peer demonstration, and training activities. *e*Health offers the convenience of autonomy over training schedules and reduced demand for outpatient visits that provides a supportive physical distancing, non-contact approach. The peer-led teleconferencing component involves experienced MWC users with specialized training and lived experience who can leverage their own experience and ability to relate as influential peers. The peer trainer conducts 3 real-time, web-based video conferencing sessions using the Microsoft (MS) Teams cloud platform via the same computer tablet. Individualized goals are negotiated, introducing wheelchair skills and management strategies, and providing an orientation to the *e*Health home program. Peer trainers monitor *e*Health training activity, which the tablet uploads to a secure website, intervening or modifying the program when required. Additional communication can be initiated between MWC user and peer trainer using a messaging function of MS Teams on the computer tablet.

## Background and Significance

**Demand for MWC use in Canada is common and growing**. In 2016, **Giesbrecht** and **Miller**[1] reported that close to 200,000 community-dwelling Canadians use a MWC; the number of wheelchair users has risen by 30% over the previous ten years. Chronic conditions, which increase with age, are often predisposing factors for use of a wheelchair, including cardiovascular disease (e.g., stroke and amputation secondary to diabetes), multiple sclerosis, arthritis, and neuromuscular diseases. An aging Canadian population suggests the number of MWC users will grow substantially in the coming years.

**Challenges to both the individual and health care system.** MWC users experience restricted participation, mobility, and quality of life. **Giesbrecht** and **Miller** recently reported that among persons with a disability, wheelchair users were more likely to experience home accessibility issues; require assistance with more activities of daily living on a more frequent basis; and have greater out-of-pocket expenses to obtain assistance.[2] In Canada, over 95% of MWC users required caregiver assistance with at least one major life activity[2] including 37% for basic mobility.[3] Restricted mobility is associated with reduced participation and social connectedness,[4] leading to isolation, stress and low self-esteem impacting quality of life.[5] Stroke survivors adjusting to MWC use identified substantial restriction in *caregivers'* social roles and increased burden of care;[6] these are typically family members or unpaid individuals. In Canada, the **annual** incidence of tips/falls is 5.2%, with 4.2% resulting in injury and 2.5% requiring an ER visit.[7] In the US, wheelchair-related accidents result in **1 death per week**.[8]

Infection prevention and control (IPC) measures are based on federal, provincial, and local epidemiological evidence tailored to the local context and based on a public health ethics framework.[69,72] The evidence-based infection prevention and control guidelines and recommendations from PHAC, including physical isolation and distancing, aimed at lowering the incidence of exposure and SARS-CoV-2 (coronavirus; COVID-19) infection, compound a MWC user's isolation and access to outpatient health care providers, transportation, community, employment, and personal support persons.[70,71,72] Telephone, web-based, or other means of telecommunication technology are recommended to be used to conduct virtual assessments and communication when in-person assessment is not necessary.[69]

**Issues related to MWC use can be redressed through MWC skills training, but typically are not.** Two recent systematic reviews of structured MWC skills training programs found them to be safe, practical and effective; [9, 10] several RCTs report significant and clinically meaningful improvement in skill capacity during inpatient rehabilitation[11, 12] and in the community.[13, 14] However, training is typically delivered by a physical or occupational therapist and requires 4 to 8 sessions; at least 10-12 hours of training is recommended to ensure safe and proficient performance.[15] During a hospital stay, when most MWCs are prescribed, competing discharge demands are often prioritized over training. **Best, Routhier** and **Miller**, in a survey of 68 Canadian rehabilitation centres, reported over half of therapists spent two hours or less on wheelchair-related training and 18% provided no training.[16] Funding for community-based services has been in decline and is insufficient to support therapist-intensive training either before or after discharge.[17, 18] Time and travel demands for MWC user and therapist make traditional training cost-prohibitive.[19] Long wait lists and inaccessibility of rehabilitation services, particularly in rural areas, further exacerbate the problem.[19, 20] In light of the SARS-CoV-2 pandemic that began in Canada in March 2020, strategies and recommendations for at-risk populations to prevent infection also complicate face-to-face delivery of health services, including MWC training. Recommendations to self-isolate and practice physical distancing, maintaining clean hands, hand rims, and wheelchair, and avoidance of in-door/out-door community settings with large crowds are among these complicating factors. The World Health Organization (WHO) states that "People with disability may also be disproportionately impacted by the outbreak because of serious disruptions to the services they rely on. The barriers experienced by people with disability can be reduced if key stakeholders take appropriate action".[73] Training is ideally delivered early in the transition to MWC use. New users (defined in the literature as < 1 year)[10] have shown significantly larger improvements with structured training; however, length of use is not correlated with skill capacity[21](Lemay et al., 2012) and comparable training benefits have been

demonstrated among experienced MWC users.[9, 10, 13, 14] Some data suggest females have lower initial self-efficacy[22, 23] and may require more practice for advanced skills[24]; however, structured training has proven beneficial for both sexes. Likewise, the *application* and *clinical benefits* of training are applicable across diagnoses. Meta-analyses of MWC skills training have demonstrated significant differences[9, 10] and a large effect size (SMD = 2.0) across studies among heterogenous participant samples, and sub-analysis found no significant interaction for diagnosis.[9, 10]

**Health care is changing and disruptive strategies are necessary**. Alternative and disruptive rehabilitation approaches are required that are clinically effective, cost-effective, COVID-19 pandemic focused, and sustainable. Two strategies demonstrate the potential to reduce burden on the healthcare system: (1) delivery in the community via *e*Health and (2) use of peer trainers. These approaches are compatible and synergistic, providing an opportunity to ameliorate training issues among MWC users in a way that is cost-effective; delivered at the most appropriate time (i.e. post-discharge) and in the most appropriate context (i.e. participant-centered, within community, mitigating COVID-19 infection risk); and in a way that is sustainable. Moreover, they integrate important theoretical precursors known to influence behaviour, in this case wheelchair use and social participation. *e*Health and peer training can cultivate psychological needs identified by Self-Determination and Social Cognitive theories.

A peer, who has experiential knowledge and similar characteristics as the target population,[25] can cultivate social connections through sharing of realized knowledge. Peer-led training is feasible to administer[26] and effectively improves wheelchair skills, self-efficacy, and satisfaction with participation.[27] Providing *e*Health training at home fosters autonomy (i.e., choice and volition), while peers enhance feelings of competence (i.e., self-efficacy) and relatedness (i.e., feeling connected).[28, 29] When these three needs are satisfied, autonomous motivation (i.e., engaging in an activity by choice) increases, driving behaviour change and maintenance, such as wheelchair use and social participation.[30] Every province and territory in Canada has implemented delivery of *e*Health,[31] providing potential partnership opportunities for TEAM Wheels. By integrating a peer-led approach with the *e*Health training program, we are leveraging two novel strategies. This is a contemporary approach to community rehabilitation with MWC users that builds on innovations we have already developed in Canada. The results of this trial will provide evidence to move forward with implementation of service at the regional health care delivery level. Furthermore, access to TEAM Wheels has the potential for future exploration in the context of rural and remote service delivery, where consumers have even greater restriction to health care access**.**

**Purpose.** Our purpose is to *evaluate the effect* of a 1-month, peer-led *e*Health training program (TEAM Wheels) on satisfaction with activity participation and related rehabilitation outcomes among individuals transitioning to MWC use, compared with current wheelchair training practice.

**Hypotheses:** We hypothesize that at post-treatment, the primary outcome of wheelchair-related participation will be significantly higher in the TEAM Wheels group and that post-treatment improvements will be retained at 6-month follow up. We also hypothesize that the TEAM Wheels program will demonstrate clinically and statistically significant differences at post-treatment and at 6-month follow up on secondary outcome measures of wheelchair-specific self-efficacy; skill capacity and performance; health-related quality of life; and objective measurement of mobility.

**Objectives:**
**Primary Objectives: Participation**

1. To measure the effect of TEAM Wheels on *satisfaction with participation* in important activities of life compared to current practice;
2. To measure retention of participation benefits at 6-months post treatment.

**Secondary Objectives: Additional Rehabilitation Outcomes**

1. To compare the effect of TEAM Wheels to current practice on wheelchair skills capacity and performance; wheelchair-specific self-efficacy; health-related quality of life; and objective measurement of wheelchair mobility.
2. To measure retention of rehabilitation outcome benefits at 6-months post treatment.

To prepare for the current project, we recently established the feasibility of delivering MWC training using both *e*Health[32] and peer-led[26, 33] strategies. An *e*Health training program was developed to allow MWC users to learn and practice wheelchair skills at home, using a 10" computer tablet mounted on their lap. The intervention involved participants attending two appointments at a rehabilitation centre with an occupational therapist, who monitored their tablet-based home training online. A feasibility randomized controlled trial (RCT) conducted by members of this research team demonstrated that the intervention could be delivered safely (i.e., no adverse events or injuries) and efficiently (i.e., no major protocol deviations).[32] Participant retention was high (94%), as was intervention group adherence (78%) to the minimum training requirements. Participants perceived the intervention as beneficial (98% agreement) and large effect sizes were found for measures of participation ($\eta^2 = 0.32$), self-efficacy ($\eta^2 = 0.22$) and wheelchair safety ($\eta^2 = 0.21$). A peer-led MWC training program was also developed and evaluated in two CIHR-funded RCTs (n=28; n=40) by members of this research team.[26, 27] Peer trainers were MWC users with established skills and specialized training who provided six 1.5-hour training sessions to less experienced MWC users in a community setting. The peer-led training program was also delivered safely (no adverse events or injuries) and efficiently (no major protocol deviations). Study retention was high (95% post-treament; 87.5% follow-up), as was adherence (95%); all intervention participants reported perceptions of benefit.[26] Large effect sizes were reported for skill capacity ($\eta^2 = 0.31$), skill performance ($\eta^2 = 0.20$), and self-efficacy ($\eta^2 = 0.35$), and a medium effect size for participation ($\eta^2 = 0.11$).[27]

These three RCTs established robust intervention protocols that can be implemented safely in a community setting. Integrating *e*Health and peer-led approaches offers considerable benefit. First, it leverages two complementary strategies with clear evidence for clinical improvements. Specifically, *e*Health training had a large effect on participation, self-efficacy, and safety while peer-led training had a large effect on MWC skill capacity, performance and self-efficacy. By merging the two approaches, we anticipate impacting all of the targeted outcomes. Second, integration addresses potential drawbacks of each individual approach. A peer trainer (i.e., less costly and more accessible) eliminates the need for an occupational therapist or rehabilitation clinician to implement and monitor the intervention. The *e*Health home program creates greater efficiency by reducing the number of in-person sessions required in the peer-led approach while increasing the opportunity for practice and repetition of skills training.

**Methods:**

**Research Design.** This RCT employs 3 sites (Vancouver, Winnipeg, Quebec City) comparing a 4-week MWC training intervention and wait-list control group among individuals transitioning to MWC use. TEAM Wheels will be delivered in the community specifically targeting new users, defined in the literature as individuals within one year of receiving their MWC.[10]

**Methods**. Participants will be randomly assigned to the TEAM Wheels or control group using a 1:1 allocation ratio between groups. To support balance between groups and masking of assignment, our statistician (Jean LeBlond, Ph.D. – Quebec City) will create a computerized randomization process with undisclosed block size. Randomization will be stratified by site to ensure comparable treatment group size (*n* ~ 18 per site). Given the potential benefits of the intervention already reported in our previous studies, control group participants will also receive the TEAM Wheels intervention following a 4-week control period and post-treatment data collection (they will be on a "wait-list" for the TEAM Wheels program and receive **no intervention** during the study period).

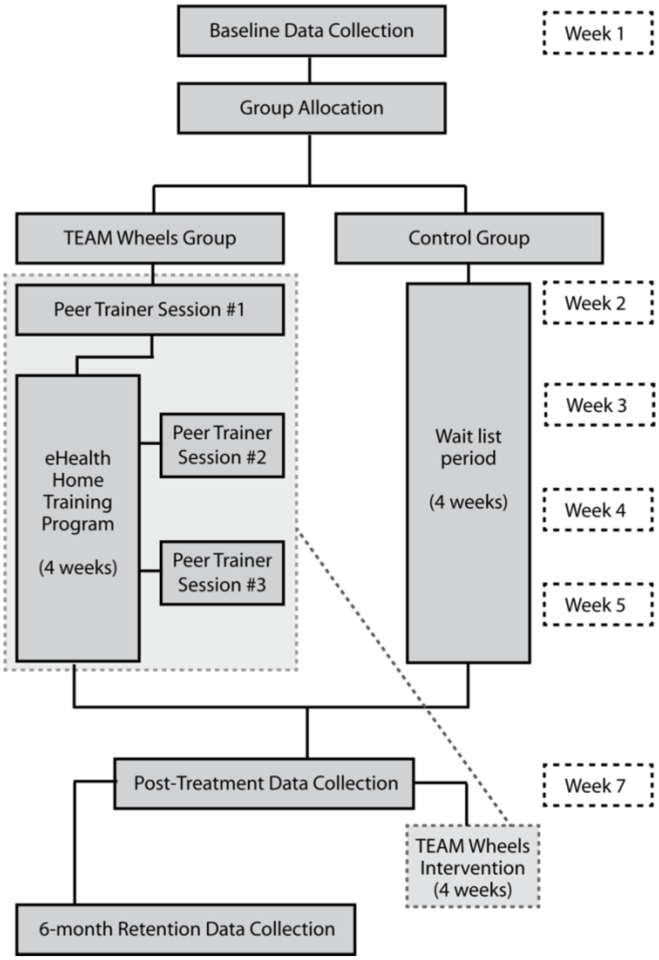


Following enrolment, baseline data will be collected and entered into a secure MS Teams database. MS Teams data storage is approved by the University of Manitoba Health Research Ethics Board (HREB) and is *Personal Health Information Act* (PHIA)/*Freedom of Information and Protection of Privacy Act* (FIPPA) compliant. The site Research Assistant (RA) will then obtain the participant's group assignment and coordinate timing for the first teleconference peer trainer session conducted via the computer tablet; for treatment group assignments this will occur as soon as is practical and for control group assignments this will be scheduled after blinded post-treatment data collection is complete. The Research Assistant will also arrange blinded follow-up (retention) data collection for treatment group participants 6 months after post-treatment data collection. Although collecting 6-month retention data for the control group would be optimal, delaying a potentially beneficial intervention and the associated risk of participant attrition make this option undesirable.

Data collection for all time periods (baseline, post-treatment, and 6-month follow-up) will be completed by the RA and entered using a laptop computer (into MS Teams). Participant confidentiality will be protected in the following manner: (1) the laptop will be encrypted and

password protected. Only the RA using the laptop/tablet will have the passwords and the laptop will be locked in their office in a locked cabinet when not in use, (2 Each participant will be assigned a unique study identification number (Study ID). A separate Master List will be created that contains only the participant's name and study ID number (saved as password protected file on internal research drive); (3) All data entered will be de-identified and coded using only the unique Study Identifiaction Number assigned to each participant, and (4) The data will be uploaded from the laptop to the MS Teams secure storage platform (in de-identified form).

Each of the three study sites (Vancouver, Winnipeg, and Quebec City) will be recruiting and collecting data for n=18 participants (for a total of N=54). The data from each sub-group will be combined for data anaysis. Hence, a tri-group data sharing agreement (DSA) will be outlined in the Informed Consent Form (ICF) and implemented; it will include the University information and Principle Investigator of each provincial team lead. De-identified data from each site's sub-group data set will be amalgamated/combined to form the complete data set for analysis. No personal (health) information will be shared across/among research teams. In addition, the fact that the scientific community, the granting agencies, and medical scientific journals are increasingly requiring that data be stored and made available for secondary review and analyses will be outlined in the ICF emphasizing that any information sent out of the University of Manitoba will not show participant's name or address, or any other identifiable personal information about them**.**

**Assessments.** Our experience in the feasibility studies,[26, 32] confirmed the pragmatic and financial challenges of attending appointments for individuals with a mobility limitation, as reported in the literature.[34-36] A Research Assistant at each site will administer the outcome measures at baseline and subsequent data collection points; participants will receive a stipend of $50. Due to the ongoing coronavirus pandemic and in line with the ever-changing guidelines and regulations, participants will be offered a variety of options for stipend payment as well as for data collection. For payment, participants will be provided with the following options:

1) A Visa or MasterCard gift card sent via postal mail or delivered to the participant's address (participant name and information would remain confidential);
2) A cheque sent directly to the participant's financial institution for deposit (the participant would agree to providing their name and financial institution address to the UM Finance Department);

For data collection, the questionnaires will always be provided to participants in advance by email, non-contact drop off, and/or post. Participants will be provided the following options for questionnaire completion:

1) The participant completes the questionnaires (except the WhOM) independently and returns them either electronically (i.e., email or text the form or image of the form) or in a self-addressed stamped envelope (SASE) by post. The RA will conduct a follow-up phone call or videoconference to confirm or correct any missing data and administer the WhOM.
2) The participant (i.e., those with internet access) completes the questionnaires (except the WhOM) online using the secure Qualtrics platform (i.e., PHIA/FIPPA compliant and HREB acceptable from personal communication). Participants receive a web link and ID

number to ensure confidentiality. The RA will conduct a follow-up phone call or videoconference to confirm or correct any missing data and administer the WhOM.

3) The RA will administer and score all of the questionnaires over the phone.
4) The RA will administer and score all of the questionnaires via teleconference using the Zoom or MS Teams platform configured for research purposes (i.e., PHIA/FIPPA compliant). The RA will "screen share" the questionnaires (either in original form or using the Qualtrics platform) and document the participant's responses.

During the coronavirus pandemic and associated restrictions, researchers will not be conducting home visits, having participants come in for visits, or meeting in public/community areas. In addition, infection prevention and control practices will be implemented. All study equipment loaned to study participants will be sanitized/disinfected prior to sending it out as well as when the equipment is returned to the research team. This will be done according to the Government of Canada recommendations for preparing/choosing hard-surface disinfectants, including (1) preparing a fresh solution using bleach with a concentration of 5% hypochlorite and combine in a 5 mL bleach: 250 mL water ratio, (2) using medical grade disinfectant wipes, (3) using an alcohol-based solution with a minimum 70% ethanol or isopropyl alcohol concentration[74,75]. The RA responsible for disinfecting the equipment will be trained in the proper handling and use of cleaning products and disinfecting/sanitizing procedures and will be provided and expected to wear the recommended personal protective equipment (PPE), including non-medical mask and gloves, during the sanitization processes. Post-treatment data will be collected at week 7 to incorporate a 1-week window for consolidation of learning following the 4-week intervention. Follow-up (retention) data will be collected from the TEAM Wheels group participants 6 months later (week 31).

**Subjects and Recruitment**. Participants are 18 years of age and older; within 12 months of receiving their first MWC; living in the community; able to propel with both arms; have sufficient cognitive and language abilities to engage with the training material; do not have a health condition that might interfere with training (e.g. cancer; surgery); and are not concurrently receiving any MWC skills training intervention. Recruitment will occur in three cities (Vancouver, Winnipeg, and Quebec City) targeting MWC users nearing hospital/rehab discharge or already living in the community. The investigators have established relationships with clinical partners in each city and experience with collaborative recruitment of MWC users.[26, 32] Active recruitment strategies, engaging health care providers rather than relying on potential participants to initiate contact, is highly effective.[37, 38] Patients are more likely to participate when recommended by a health care provider.[39] To this end, a number of tailored strategies to support effective and efficient participant recruitment will be employed, including:

(1) Allied health professionals in facilities at each site (eight potential sites) will approach patients who meet the eligibility criteria to explain the TEAM Wheels program and obtain consent for investigator contact; clinical champions have already been identified and provided letters of support. *The Permission to Contact (PTC) Form-TEAM Wheels-Hospital/Institution Sites*; *Version #2; September, 2020* will be used at each hospital/site to collect this information (either as paper-based hard copies or verbally depending on each hospital's preferred method based on COVID-19 prevention protocols). The logos on this PTC Form will use the relevant logo to match the site involved (i.e. University of Manitoba & St. Boniface Hospital logos for St. Boniface Hospital Site; University of Manitoba & Health Sciences Centre logos for Health Sciences Centre

site). The Research Assistant will follow up with patients who have provided their consent to be contacted via telephone and/or email to discuss the study and ascertain their interest in study participation. In those circumstances where the hospital site/OT clinic is reducing paper use as part of their mitigation strategy to reduce the risk of COVID-19 infection, OTs/clinic staff will describe the study briefly and verbally read the PTC form to eligible participants, inquire about interest in obtaining further information from research staff, and collect the patient's name and contact information if they provide their verbal consent to be contacted to learn more about the study and their potential participation. The Research Assistant will work with each hospital OT/clinic staff unit to create and implement a tailored information transfer process that facilitates providing the Research Assistant with the names and contact information the OTs/clinic staff have obtained from patients consenting to be contacted about the study. This process will maintain patient health information security and safety as it will be done over the telephone or through a secure Zoom or MS Teams teleconference.

(2) Additional recruitment will be conducted in the community through public advertisements; advocacy and consumer groups; wheelchair vendors; and outpatient Community Therapy Services providers. Specifically, a collaborative partnership between the TEAM Wheels research team and Manitoba Possible's Wheelchair Program (MWP) has been established that will facilitate a more effective recruitment strategy. This will include: (1) distribution of a *MWP-TEAM Wheels Permission to Contact Form (*Version #3, April-2021) to each MWP Wheelchair requisition applicant (paper-based and/or electronic) meeting the inclusion criteria (determined by MB Possible staff). The MWP-TEAM Wheels Permission to Contact Form is opt-in, therefore only those clients interested in learning more about the TEAM Wheels research study and their possible participation, will complete and return the form (or contact research staff via telephone and/or email as outlined in the form). Completed forms returned by mail will be collected by MWP and provided to the TEAM Wheels research team for follow-up based on the process outlined. (2) A video outlining the research study that is based on the HREB approved TEAM Wheels pamphlet and poster will be created and posted on the MWP website and social media platforms (LinkedIn, Facebook, Twitter, Instagram, and YouTube), and (3) a list of MWP clients meeting the TEAM Wheels research study eligibility criteria, from the previous year (beginning in May, 2021 and onwards until all 18 participants have been recruited) will be compiled by MWP and each individual on the list, mailed the PTC form directly from MWP. The list will be composed of the name and mailing address of the clients to whom the MWP will mail a MWP-TEAM Wheels Permission to Contact Form (Version #3; April, 2021), along with a postage paid return envelope. This has been approved by the MWP. Implementation of the MWP-TEAM Wheels Permission to Contact Form through the MWP and list of clients from the previous year will facilitate more efficient and effective participant recruitment. This approach/strategy will streamline the process of recruitment by supporting contact at a key point in the distribution of wheelchairs to clients transitioning to wheelchair use in Winnipeg and Manitoba. This strategy will also decrease the burden on the OTs and clinical staff (from the hospital sites) to initiate and coordinate obtaining permission to contact from clients/patients while in hospital and having to contact TEAM Wheels researchers to coordinate client contact and future follow-up. We have seen that the former process results in insufficient participant enrollment and requires substantially more human resources and time to coordinate and implement. The video to be created and posted on the MWP website is an extension of the HREB approved advertising (pamphlet and poster) meant to inform both clinicians and potential participants about the TEAM Wheels study and potential benefits from collaboration

(clinicians) and participation (clients). In addition, MWP will add the TEAM Wheels research study PTC, pamphlet/brochure, poster, and social media videos and recruitment information sketches to their website.

Social media-based recruitment will occur via informational and multimedia (image and video based) posts on the platforms mentioned above (LinkedIn, Twitter, YouTube, Instagram, and Facebook) and through relevant groups/pages within these platforms. All images and videos used for recruitment purposes will be developed to provide clear and understandable information about the study and based on previously approved HREB advertising/recruitment sources (poster, brochure, and TEAM Wheels App.) supporting the opportunity to reach as many eligible individuals as possible for potential recruitment/participation. Some of the graphics will be developed using cartoon style and infographic software and will be featured in both French and English. Social media posts will include contact information for the local study site PI/research personnel. In addition, the social media posts will let potential participants know that they can reach out via the direct message function on the respective media platform to the local study site PI/research personnel with any questions or concerns, which will be followed by additional conversations via secure methods (telephone, MS Teams, or Zoom).

All potential study participants will be provided with a copy of the *Research Participant Information and Consent Form* (Version #6; April, 2021) (i.e. via post or email). The RA will review the ICF with potential participants and answer any questions they have via telephone and/or video-conferencing (Zoom or MS Teams in secure PHIA/FIPPA compliant configuration) in order to facilitate understanding and completion of the ICF.

Participants will be provided the following options to confirm consent:

1) Return the signed ICF using a self-addressed stamped envelope (SASE) through regular mail;
2) Return a signed image (e.g., pdf or photo) of their completed ICF;
3) Audio recording obtained/captured by the RA reviewing the ICF by videoconference and confirming that they understand, have had their questions answered, and have consented to participate; or
4) Provide consent using an online version of the ICF via the secure Qualtrics (or MS Teams) platform (PHIA/FIPPA compliant).

**Outcome measures.** At baseline, descriptive characteristics including age, sex, gender, marital status, highest level of education, primary diagnosis related to MWC use, and length of time using the MWC will be collected. Five outcome measures (available in both French and English) will be administered at baseline**,** post-treatment, and (for treatment group participants) at 6-month follow-up. The RA will complete the questionnaires with the participant via telephone or Zoom/MS Teams teleconference. The participant will be provided with the questionnaire in advance, via post or email. The RA will collect the data by telephone or teleconference and (subsequently) enter it into a password protected Excel file in the MS Teams database.

*Satisfaction with Participation (primary outcome).* The International Classification of Functioning, Disability and Health (ICF) identifies participation as "involvement in a life situation,"[41] including the subjective experience and performance of important activities and roles. The Wheelchair Outcome Measure (WhOM) is a patient-oriented measure of satisfaction with participation, specifically related to relevant activities inside and outside of the home. The WhOM

uses user-specific activities, addressing potential sex and gender differences in viewing participation.[42] Two published literature reviews identify the WhOM as the most client-centred and psychometrically robust tool among participation outcomes for wheelchair users.[43, 44] The WhOM is administered as a semi-structured interview. Respondents identify 10 activities (5 performed inside and 5 performed outside of the home), rating their satisfaction with performance on an 11-point scale (0-10) for each activity. The RA will complete the WhOM with the participant via telephone or Zoom/MS Teams teleconference. The participant will be provided with the WhOM form along with the other questionnaires in advance, via post or email. The RA will collect the data by telephone or teleconference and (subsequently) enter it into the MS Teams database. The WhOM demonstrates good reliability (Test-retest ICC = 0.83-0.88; Inter-rater ICC = 0.90-0.91) and validity (correlations with LIFE-H: $r_s$ = 0.3-0.5) in use among individuals with spinal cord injury[45] and older adults (Test-retest ICC = 0.77–1.00; correlation with QUEST $r_s$ = 0.36 - 0.45).[46](Auger et al., 2010) A change of 1.6 points has been identified as clinically meaningful.[45] Combined results from our feasibility studies found a large effect size (*d* = 0.93; 95%CI 0.54, 1.33), with mean improvement of 2.3 points. WhOM activities identified by participants (but not the scores) will be shared with their peer trainer to facilitate tailoring of the TEAM Wheels program.

*MWC Skills Capacity and Performance*. To address environmental barriers, MWC users must learn specific mobility skills such as managing inclines, thresholds, and doorways. The Wheelchair Skills Test - Questionnaire v 5.0 (WST-Q) evaluates 33 discrete skills; respondents rate their *capacity* (i.e. ability) with each skill as well as *performance frequency*. Capacity is rated as Very well (3), Yes (2), Partially (1), or No (0); performance is rated as Always (3), Usually (2), Occasionally (1), or Never (0). Total skill Capacity (0-100%) and Performance (0-100%) scores are calculated. Two systematic reviews of MWC skill outcome measures confirm the WST has the strongest psychometric properties and has been used most extensively in clinical trials.[47, 48] The WST has demonstrated excellent reliability for test-retest (ICC=0.90), intra-rater (ICC=0.96), and inter-rater (ICC=0.97) administration.[49](Kirby et al., 2004) The WST-Q correlates highly with the WST objective testing version (*r*=0.89), with much lower administration burden (~10 minutes vs 30 minutes) and can be used to evaluate both capacity and performance.[50]

*Wheelchair-specific Self-efficacy*. Self-efficacy, or *belief* in one's ability to use a wheelchair,[51] is a significant determinant of participation frequency and mediator of skill capacity.[52] The Wheelchair Use Confidence Scale-Short Form (WheelCon-SF) is a self-report questionnaire with 21 statements related to confidence using a wheelchair in various activities and environments. Items are rated on a scale from 0 ("not confident") to 10 ("completely confident"), providing a total mean score (0-10)[23]. The WheelCon-SF is more responsive than the original 65-item version;[23] a recent study reported high test-retest reliability (ICC=0.98) and internal consistency (Cronbach's alpha = 0.95).[53] Results from our feasibility studies found a statistically significant difference in self-efficacy (large effect size: $\eta^2 = 0.28$), with the treatment group improvement exceeding the threshold for meaningful change.[22]

*Health-Related Quality of Life*. Health utility measurement is useful when evaluating the impact of rehabilitation interventions. National guidelines for healthcare economic analyses strongly advocate the use of a validated measure of *health-related quality of lif*e (HRQL), which can be converted to quality-adjusted life years (QALY) gained to fully inform funding decisions.[54] ~~The~~ The SF-36E (enabled version) is a measure of health status commonly used in health economics as a variable in the QALY calculation to determine the cost-effectiveness of a health care

intervention/treatment[55a, 55b]. The SF-36 is a 36-item patient-reported questionnaire that covers eight health domains: physical functioning (10 items), bodily pain (2 items), role limitations due to physical health problems (4 items), role limitations due to personal or emotional problems (4 items), emotional well-being (5 items), social functioning (2 items), energy/fatigue (4 items), and general health perceptions (5 items). The SF-36E is adapted for mobility-impaired individuals (the word "go" replaces "walk" and "climb") and has been demonstrated to successfully measure performance among mobility-impaired individuals, including wheelchair users.[55c]

The multiple-attribute utility scale is scored from -0.36 to 1.00, with higher scores reflected better health and quality of life. Acceptable test-retest reliability was found among patients recovering from hip fracture (ICC = 0.72).[54] A change score of 0.03 is considered representative of a minimally important difference;[56, 57] results from our feasibility study identified a change score of 0.17, reflecting a medium effect size.

*Objective Wheelchair Mobility*. To triangulate measurement of improvement in mobility and community engagement, accelerometry data (Actigraph™, Pensacola FL) will be used to objectively measure MWC activation over a 10-day period at baseline, post-treatment, and follow-up data collection points.[58] The Actigraph is attached to a rear wheel on the MWC; a battery provides up to 14 days of passive data collection without impeding MWC operation. Data loggers have demonstrated valid and reliable[59, 60] measurement of MWC movement, with accuracy beyond 90%.[59] A tri-axial accelerometer records acceleration forces during MWC use, downloadable as a .csv file. Algorithms developed by our team convert data into total and mean values of distance, speed, and 'bout' frequency (meaningful transitions between functional activities[60]), parameters that reflect mobility patterns and activity of MWC users which we expect to change as a result of improvements in skill and participation. The GT3X Actigraph will be turned on, assembled in a tailored wheelchair holder, disinfected, and mailed or delivered (using non-contact method) to each participant, along with a return box and shipping label. A user-friendly, step by step instruction sheet will be provided to facilitate participants installing the actigraph onto their wheelchair. If necessary, the RA can assist participants with troubleshooting actigraph installation via telephone or video communication (Zoom/MS Teams). Actigraphs will be returned using the provided box with shipping label. Once returned, all equipment will be disinfected with hospital-grade disinfectant according to standard protocols (registered in Canada with a Drug Identification Number (DIN), and labelled as effective for both enveloped and non-enveloped viruses) before being sent out to the next participant.[71] All safety measures and protocols, including personal protective equipment (PPE), will be used by research staff disinfecting the study-related equipment.

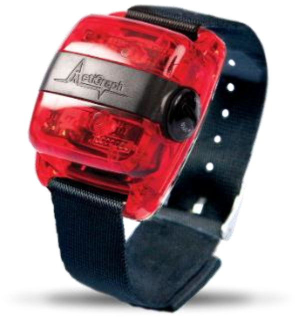


*Mobility Research Registry*. The University of Manitoba College of Rehabilitation Sciences, Occupational Therapy Program has created an HREB approved Mobility Research Registry (HS22050 (H2018:307) that includes a *Participant Information and Consent Form for permission to be contacted for future mobility-related research at the College of Rehabilitation Sciences (Version #2; October, 2019)*. Study participants at the Winnipeg site will be provided with the form and asked if they would like to complete it after being given an explanation of the Registry and its purpose as well as the Permission to Contact Form (according to the HS22050 HREB protocol). Participants have to opt in or out. Those participants opting in will complete the form which will be handled in accordance with the HREB approved Mobility Research Registry

processes and protocol. Data collection is completed over the telephone with the RA, utilizes paper-based data collection, with data entered into two separate Excel spreadsheets (password protected and not connected to a network).

**Intervention**. As already detailed, the intervention integrates two effective community-based approaches developed to improve mobility and participation through MWC training. A more detailed description of the treatment and control interventions follows.

**Treatment Group**. The treatment group receives the TEAM Wheels program over a 4-week period. An initial meeting (Session 1) is pre-arranged by the RA via telephone or video-conference using a secure Zoom/MS Teams platform (PHIA/FIPPA compliant). The peer trainer is an experienced MWC user trained to deliver the TEAM Wheels program. At least 2 peers will be hired and trained at each site to offer multiple trainer attributes; we will intentionally include a male and female, one being at least 50 years of age. Participants will pre-select a peer trainer based on a biosketch to optimize training effect (e.g., preference for age and sex factors); comparability in age has been identified as preferential among older adults[61] and influential to self-efficacy.[28]

Prior to the first session the RA will pre-configure the tablet and Wi-Fi hotspot device. All required equipment (i.e., tablet; Wi-Fi hotspot; tablet holder; gloves; spotter strap; instruction sheets for tablet set up, MS Teams (and Zoom) teleconference set-up, and actigraph attachment; and user manuals) will be disinfected/sanitized and couriered or delivered (via non-contact methods if preferred) by the RA to the participant's home. The RA will contact the participant by telephone or secure Zoom/MS Teams platform to assist with them with set-up, ensure they are able to connect to the teleconference application, and have a link to initiate their first session teleconference. The RA will be available to assist the participant with troubleshooting; participants will be provided with an office telephone number to reach the RA and that will be checked regularly.

During Session #1, the peer trainer and participant are introduced and collaboratively identify targeted outcomes related to participation, mobility and MWC management; informed by activities identified on the Wheelchair Outcome Measure (WhOM)[45, 62] administered at baseline. The peer trainer tailors the program to address the participant's personal goals and current skill proficiency[63] (session content; specific skills/content in *e*Health program). The trainer provides an orientation to the tablet and *e*Health home training application (TEAM Wheels app), explaining the application functions and watching the first video called "how to use this tablet app" together with the participant. The peer trainer will then ask the participant to navigate through the program components using the "screen share" function of Zoom/MS Teams to confirm the participant is successful.

The peer trainer will also provide training in basic skills and safety. The teleconferencing application will be used to both watch and demonstrate skill performance. Wherever possible, care providers (within the participants COVID-19 bubble and according to MB Health regulations at the time) will be included in sessions to promote participant adherence, engagement and safety. Study participants will be asked to have a spotter available during each session (e.g., a family member, friend or caregiver within their COVID-19 bubble); the peer trainer will provide a demonstration of how to safely "spot" either using video included in the home training application or by demonstration on the teleconference application. If no spotter is available, participants will be cautioned about which skills might create a high risk for tipping and to only attempt these when a spotter is present. The participant and peer negotiate appointments for Sessions #2 and #3, which will also be conducted via tele-conference (Zoom/MS Teams). The peer trainer facilitates SMART

goal setting,[64] MWC skill instruction, education about potential physical and subjective barriers, and strategies to overcome challenging situations.

Following Session #1, participants engage in 4 weeks of training with the *e*Health home program. They are instructed to practice for 75 to 150 minutes per week. Consistent with motor learning principles, we encourage training in 15-30 minute blocks 1-2 times per day, 3-5 days per week.[65]

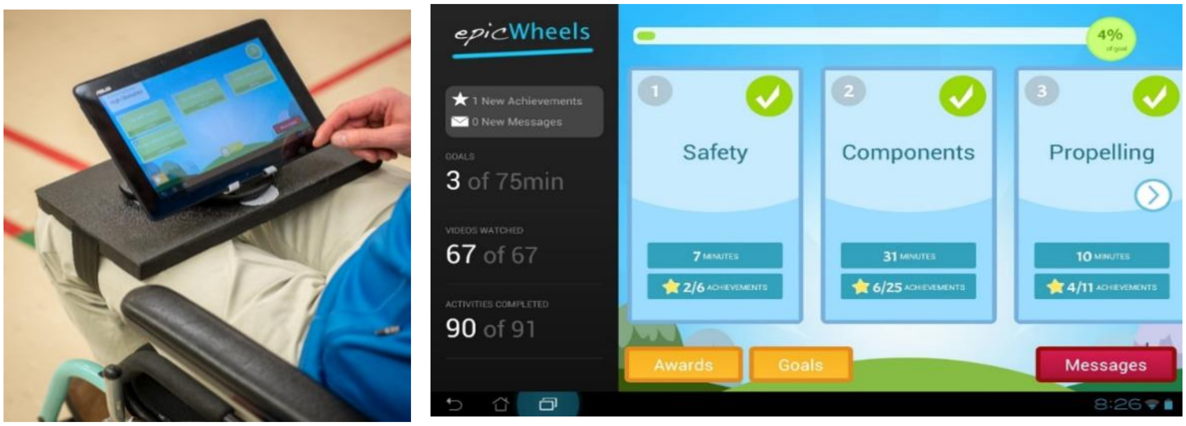


***e*Health Home Training Program: Mounting system for in-chair use and software**

The tablet displays feedback to the participant on training activity and weekly progress relative to these targets. The tablet software incorporates over 200 video-based training elements including instruction, peer demonstration, and practice activities and games. The tablet software records all home training activity and uploads this data to a secure server. For participants without Internet access, a portable Wi-Fi hotspot device is provided. The peer trainer monitors participant progress online and may adapt the program, or follow-up with the participant, based on their progress data. Participants and peer trainers can also exchange messages when necessary, using the *e*Health tablet (participant) and The Zoom/MS Teams platforms or email.

**Control Group**. The control group receives no specific intervention over the course of the 4-week period; this reflects "usual practice" or the typical experience of a MWC user in their provincial context. Participants are placed on a wait-list and receive the TEAM Wheels program, as described, following completion of the study (i.e. after post-treatment data collection from the Intervention group). The site Research Assistant will make contact with control group participants at the end of weeks 2 and 4 during the study period to deter attrition/drop-out. When contact is made at week 4, the Research Assistant will schedule an appointment for post-treatment data collection (week 7). Any formal MWC training received during the wait-list period will be documented for potential post-hoc analysis as a confounding variable; however, research evidence[16] and the investigators' clinical experience confirm that in all 3 provinces formal training is not provided once MWC users are discharged from hospital.

**Data Analysis.** After testing for assumptions and outliers,[66] between-groups (intervention vs control) comparison of change over time will be conducted using Generalized Linear Modeling (GLM) for participation; MWC skill capacity and performance; self-efficacy; health-related quality of life; and objective MWC mobility parameters ($\alpha = 0.05$).[67] Variables previously identified in the literature as relevant covariates (i.e. age and function as measured by baseline WST-C) will be controlled for. Model fit ($R^2$), regression coefficients ($CI_{95\%}$), and effect sizes ($F^2$)

will be assessed with Maximum Likelihood methods. Data will initially be disaggregated by sex and gender; if analyses prove non-significant, the data will be reaggregated. A repeated-measures ANOVA will be used to evaluate retention of outcomes at follow-up for treatment group participants. Additional post-hoc sub-analyses will investigate the impact of site (using 2-way ANOVA) and length of MWC use covariate (using ANCOVA), although the study is powered only for the primary analysis. Descriptive data will report on recruitment by sex, relative to prevalence data for MWC use[1] to inform generalizability of findings and health services equity balance.[68]

**Sample Size.** The combined results of our feasibility studies found a large effect size ($f$ = 0.47) for the WhOM and correlation between measures ($R^2$) was 0.63. Using a comparable effect size ($f$ = 0.45) and measure correlation (0.6), α = 0.05, and 90% power for a between-factors ANOVA repeated measures design, a sample size of 42 is required (G*Power v.3.1.9.2). Adjusting for 20% loss to attrition, a total sample size of 54 was determined (i.e. 27 per group; intervention and control). We anticipate recruiting 1-2 participants/per site/per month, estimating 12 months required to recruit the sample. Incorporating the treatment and 6-month follow-up periods, the maximum data collection period should be 19 months. While we have built in a 20% attrition rate, our feasibility studies reported 5-10% drop-out rates,[26, 32](Best et al., 2017; Giesbrecht & Miller, 2017) and with the provision of the TEAM Wheels program to control group participants after data collection, we anticipate little attrition.

**Study feasibility:**

**Dr. Ed Giesbrecht** is an occupational therapist and Associate Professor at the University of Manitoba. His research program focuses on MWC mobility interventions and participation, and developed the *e*Health training program content. He will be responsible for grant administration, implementation of updated *e*Health content, site lead for Winnipeg, and be involved in data analysis and KT activities. **Dr. William Miller** is a Professor at the University of British Columbia, his research program addresses wheelchair mobility and outcome measurement. He previously led a CIHR team grant addressing power mobility innovations for older adults. He has expertise in epidemiology and clinical trial design, having led over 6 RCTs including 2 pan Canada multi-site trials. He will provide leadership on multi-site operational issues, serve as site lead for Vancouver, and be involved in data analysis and KT activities. **Dr. Francois Routhier** is a rehabilitation engineer, Associate Professor in Rehabilitation at Université Laval and researcher at the Centre for Interdisciplinary Research in Rehabilitation and Social integration (CIRRIS), with expertise in wheelchair mobility, training, and outcome measurement. He is a research scholar of the *Fonds de la recherche du Québec - Santé* since 2013. He has extensive experience translating outcome measures into French and led or participated in numerous clinical trials (5 RCTs, 2 pre-post and 5 longitudinal trials). He will oversee the *e*Health upgrades, translation process, co-lead the Quebec City site, and be involved in data analysis and KT activities. **Dr. Krista Best** is a kinesiologist, Research Scientist at CIRRIS and Adjunct Professor in Rehabilitation at Université Laval, with extensive expertise in skills training and self-management strategies. She will oversee integration and training of peer-trainers, co-lead the project at the Quebec City site, and be involved in data analysis and KT activities at study end. **Dr. Maude Laberge** is an Assistant professor in the Department of Operations and Decision Systems at Université Laval. She specializes in health services research, primary care, performance measurement, health economics, quality management, and indicator development. She will oversee the development of study-specific outcome measures used to conduct a cost effectiveness analysis of the TEAM Wheels program.

**Collaborators and Team Members**:

**Dr. Jean LeBlond** is a biostatistician at CIRRIS and project collaborator, providing consultation with data analysis as well as configuring the study randomization process. A computer engineer at CIRRIS will be hired to provide maintenance and support for the study, and conduct data reduction and analysis for the Actigraph data. An Advisory Committee includes several stakeholders. **Ms. Cherry Nixdorf** (OT, Health Sciences Centre, Winnipeg) is a knowledge user from Winnipeg. **Andrea Lavoie** (Quebec City) is a MWC user and peer-trainer. **Ms. Liz Loewen** is Director and Information Solution Officer of Coordination of Care for Manitoba eHealth (Health Manitoba) and provides expertise from the perspective of health care systems delivery, implementation, and KT.

**Study Sites**.

The three study sites offer varied provincial approaches to MWC procurement as well as differences in geography, culture and climate, and provincial COVID-19 recommendations and guidelines (Phase of restrictions) to prevent infection. This diversity allows TEAM Wheels delivery in multiple contexts; site-specific post-hoc analyses may provide additional insights. Sites are major urban centres with access to a large number of MWC users. In Manitoba, approximately 1800 MWCs were distributed to first-time users in 2016, primarily within Winnipeg (pop ~700,000). The population of Quebec City is comparable and Vancouver is over three times as large, providing a sizable pool of potential participants.

**Potential Challenges**:

It will be critical that appropriate peer trainers are in place. We already have employment relationships in place with several MWC users at each site, including several individuals previously employed as peer trainers.[26, 27] Our relationships with local advocacy/disability organizations will also be leveraged, including the Canadian Paraplegic Association (now SCI-MB) and Manitoba Possible (formerly, Society for Manitobans with Disabilities) in Manitoba. Participant recruitment is always a concern; the team is implementing contingencies based on experiences from our feasibility studies. We are using active strategies[37-39] including recruitment in-hospital; utilizing clinicians to gain early access and advocate; providing stipends for data collection; delivering the intervention in the context of the COVID-19 pandemic via eHealth in the community; and funding for on-site clinical champions. The cost of tablet purchase, damage and loss should be considered, although no tablets were damaged or lost in our feasibility RCT. Innovations in commercial tablets since the previous feasibility study provide an opportunity for the team to explore potential tablet options that are less expensive, and once effectiveness is demonstrated next steps will include exploring user devices and downloadable options.

**Timeline**.

The project was originally planned to be conducted over 3 years, between October 2019 and September 2022 (see figure). However, with the unexpected introduction of the COVID pandemic, this timeline will need to be extended. The need to revise the intervention to a completely eHealth delivery format, combined with provincial health care and university restrictions around recruitment and implementation of community-based intervention research, have delayed both preparation for, and initiation of, this study. We currently anticipate beginning recruitment in the spring 2021, completion of data collection in summer of ~~2022~~ 2023, and analyses/KT activities in spring 2024.

| YEAR 1 | | | | YEAR 2 | | | | YEAR 3 | | | |
|---|---|---|---|---|---|---|---|---|---|---|---|
| Q1 | Q2 | Q3 | Q4 | Q1 | Q2 | Q3 | Q4 | Q1 | Q2 | Q3 | Q4 |
| Ethics | | | | | | | | | | | |
| Update software | | | | | | | | | | | |
| Translate materials | | | | | | | | | | | |
| | | | | Recruit Subjects | | | | | | | |
| | | | | Data Collection | | | | | | | |
| | | | | Deliver TEAM Wheels to Control Group | | | | | | | |
| | | | | | | Follow-up Data | | | | | |
| | | | | | | | | Analyses | | | |
| | | | | | | | | | | KT Activities | |

## Knowledge translation:

The Advisory Committee will participate in teleconference meetings a minimum of every six months throughout the project to provide input and feedback, to ensure we address issues related to MWC users, clinicians, trainers and health systems. At study's end, we will hold a half-day teleconference with the committee to determine the most effective strategies and partners for pursuing systemic implementation of TEAM Wheels. The team will also disseminate project outcomes with clinical and provincial health care partners at each site, and propose implementation strategies for scaling the program at a municipal or provincial level. MWC users, clinicians and academic researchers will be targeted in our knowledge translation activities. **MWC users** are the ultimate beneficiaries of the TEAM Wheel program, with increased mobility and confidence to support social participation. We will distribute a 1-page summary infographic to study participants and disseminate it publicly through social media. **Clinicians** and **administrators** will also be interested in evidence for a MWC training program that can reduce clinical burden; we will present an informational workshop to MWC users, clinicians and provincial health care partners using our established clinical collaborations. We will reach **academic researchers** by presenting findings at four national and international conferences (e.g., American Congress on Rehabilitation Medicine; International Seating Symposium) and publish a minimum of two manuscripts in high impact and open access peer reviewed journals (e.g., Archives of Physical Medicine and Rehabilitation). Finally, we will pursue a **CIHR Planning and Dissemination Grant** to disseminate findings and explore potential delivery strategies identified with the Advisory Committee, including delivery in rural and remote locations, partnering with local wheelchair users as peer-trainers.

## References

1. Smith EM, Giesbrecht EM, Mortenson WB, et al. Prevalence of Wheelchair and Scooter Use Among Community-Dwelling Canadians. *Physical Therapy* 2016; 96: 1135-1142. DOI: 10.2522/ptj.20150574.
2. Giesbrecht EM, Smith EM, Mortenson WB, et al. Needs for mobility devices, home modifications and personal assistance among Canadians with disabilities. *Health Reports* 2017; 28: 9-15.
3. Shields M. Use of wheelchairs and other mobility support devices. *Health Reports* 2004; 15: 37-41.

4. Finlayson M and van Denend T. Experiencing the loss of mobility: perspectives of older adults with MS. *Disability and Rehabilitation* 2003; 25: 1168-1180. DOI: 10.1080/09638280310001596180.
5. Simpson RC, LoPresti EF and Cooper RA. How many people would benefit from a smart wheelchair? *Journal of Rehabilitation Research and Development* 2008; 45: 53-72.
6. Laliberte Rudman D, Hebert D and Reid D. Living in a restricted occupational world: The occupational experiences of stroke survivors who are wheelchair users and their caregivers. *Canadian Journal of Occupational Therapy* 2006; 73: 141-152.
7. Kirby RL, Ackroyd-Stolarz SA, Brown MG, et al. Wheelchair-related accidents caused by tips and falls among noninstitutionalized users of manually propelled wheelchairs in Nova Scotia. *American Journal of Physical Medicine and Rehabilitation* 1994; 73: 319-330.
8. Gavin-Dreschnack D, Nelson A, Fitzgerald S, et al. Wheelchair-related falls: current evidence and directions for improved quality care. *Journal of Nursing Care Quality* 2005; 20: 119-127.
9. Tu C, Liu L, Wang W, et al. Effectiveness and safety of wheelchair skills training program in improving the wheelchair skills capacity: a systematic review. *Clinical Rehabilitation* 2017: 1-10. DOI: 10.1177/0269215517712043.
10. Keeler L, Kirby RL, Parker K, et al. Effectiveness of the Wheelchair Skills Training Program: a systematic review and meta-analysis. *Disability and Rehabilitation: Assistive Technology* 2018: 1-19. DOI: 10.1080/17483107.2018.1456566.
11. MacPhee AH, Kirby RL, Coolen AL, et al. Wheelchair skills training program: a randomized clinical trial of wheelchair users undergoing initial rehabilitation. *Archives of Physical Medicine and Rehabilitation* 2004; 85: 41-50. DOI: 10.1016/s0003-9993(03)00364-2.
12. Worobey LA, Kirby RL, Heinemann AW, et al. Effectiveness of Group Wheelchair Skills Training for People With Spinal Cord Injury: A Randomized Controlled Trial. *Archives of Physical Medicine and Rehabilitation* 2016; 97: 1777-1784.e1773. DOI: 10.1016/j.apmr.2016.04.006.
13. Best KL, Kirby RL, Smith C, et al. Wheelchair skills training for community-based manual wheelchair users: a randomized controlled trial. *Archives of Physical Medicine and Rehabilitation* 2005; 86: 2316-2323. DOI: 10.1016/j.apmr.2005.07.300.
14. Ozturk A and Ucsular FD. Effectiveness of a wheelchair skills training programme for community-living users of manual wheelchairs in Turkey: a randomized controlled trial. *Clinical Rehabilitation* 2011; 25: 416-424. DOI: 10.1177/0269215510386979.
15. Kirby RL. *Wheelchair skills assessment and training*. Boca Raton, FL: CRC Press, Taylor & Francis Group, 2017.
16. Best KL, Routhier F and Miller WC. A description of manual wheelchair skills training: current practices in Canadian rehabilitation centers. *Disability and Rehabilitation: Assistive Technology* 2015; 10: 393-400. DOI: 10.3109/17483107.2014.907367.
17. Gill TM, Baker DI, Gottschalk M, et al. A program to prevent functional decline in physically frail, elderly persons who live at home. *New England Journal of Medicine* 2002; 347: 1068-1074. DOI: 10.1056/NEJMoa020423.
18. Tousignant M, Dubuc N, Hebert R, et al. Home-care programmes for older adults with disabilities in Canada: how can we assess the adequacy of services provided compared with the needs of users? *Health and Social Care in the Community* 2007; 15: 1-7. DOI: 10.1111/j.1365-2524.2006.00645.x10.1111/j.1365-2524.2006.00645.x.

19. Sanford JA, Griffiths PC, Richardson P, et al. The effects of in-home rehabilitation on task self-efficacy in mobility-impaired adults: A randomized clinical trial. *Journal of the American Geriatrics Society* 2006; 54: 1641-1648.
20. Tousignant M, Boissy P, Corriveau H, et al. In home telerehabilitation for older adults after discharge from an acute hospital or rehabilitation unit: A proof-of-concept study and costs estimation. *Disabil Rehabil Assist Technol* 2006; 1: 209-216. 2006/09/01.
21. Lemay V, Routhier F, Noreau L, et al. Relationships between wheelchair skills, wheelchair mobility and level of injury in individuals with spinal cord injury. *Spinal Cord* 2012; 50: 37-41. DOI: 10.1038/sc.2011.98.
22. Giesbrecht EM and Miller WC. Clinical benefits of an mHealth wheelchair skills training program for older adults. *Archives of Physical Medicine and Rehabilitation* 2016; 97: e80. DOI: 10.1016/j.apmr.2016.08.245.
23. Sakakibara BM, Miller WC and Rushton PW. Rasch analyses of the wheelchair use confidence scale. *Archives of Physical Medicine and Rehabilitation* 2015; 96: 1036-1044. DOI: 10.1016/j.apmr.2014.11.005.
24. Kirby RL, Gillis DJ, Boudreau AL, et al. Effect of a high-rolling-resistance training method on the success rate and time required to learn the wheelchair wheelie skill: a randomized controlled trial. *Am J Phys Med Rehabil* 2008; 87: 204-211; quiz 212-204. 2008/02/22. DOI: 10.1097/PHM.0b013e318164aa27.
25. Bratter B and Freeman E. The maturing of peer counselling. *Generations* 1990; 14: 49-52.
26. Best KL, Miller WC, Routhier F, et al. Feasibility of the trial procedures for a randomized controlled trial of a community-based peer-led wheelchair training program for older adults. *Pilot and Feasibility Studies* 2017; 4: 18. DOI: 10.1186/s40814-017-0158-3.
27. Best KL, Miller WC, Huston G, et al. Pilot Study of a Peer-Led Wheelchair Training Program to Improve Self-Efficacy Using a Manual Wheelchair: A Randomized Controlled Trial. *Archives of Physical Medicine and Rehabilitation* 2016; 97: 37-44. DOI: 10.1016/j.apmr.2015.08.425.
28. Bandura A. *Self-efficacy: the exercise of control*. New York: W. H. Freeman and Company, 1997.
29. Ryan R and Deci E. Self-determination theory and the relatedness of intrinsic motivation, social development, and well-being. *American Psychologist* 2000; 55: 68-78.
30. Wilson P, Rodgers W, Blanchard C, et al. The relationship between psychological needs, self-determined motivation, exercise attitudes and physical fitness. *Journal of Applied Social Psychology* 2003; 33: 2373-2392.
31. Atkinson M. eHealth in Canada: current trends and future challenges. Ottawa: Information and Communications Technology Council, 2009.
32. Giesbrecht EM and Miller WC. A randomized control trial feasibility evaluation of an mHealth intervention for wheelchair skill training among middle-aged and older adults. *PeerJ* 2017; Oct 5: e3879. DOI: 10.7717/peerj.3879.
33. Best KL, Miller WC, Routhier F, et al. Feasibility of the Wheelchair Self-Efficacy Enhanced for Use (WheelSeeU) training program: study protocol for a randomized controlled trial. *Canadian Journal of Occupational Therapy* 2016; 81: 308-319. DOI: 10.1177/000841741456743.
34. Blanton S, Morris DM, Prettyman MG, et al. Lessons learned in participant recruitment and retention: the EXCITE trial. *Physical Therapy* 2006; 86: 1520-1533. DOI: 10.2522/ptj.20060091.

35. Gomes R, Michaelsen S, Rodrigues L, et al. Scientific research with individual post stroke: difficulties in recruitment, allocation and adherence on two different protocols of physiotherapy intervention. *Fisioterapia e Pesquisa* 2015; 22: 34-40. DOI: 10.590/1809-2950/13111022012015.
36. McMurdo ME, Roberts H, Parker S, et al. Improving recruitment of older people to research through good practice. *Age and Ageing* 2011; 40: 659-665. DOI: 10.1093/ageing/afr115.
37. Page SJ and Persch AC. Recruitment, retention, and blinding in clinical trials. *American Journal of Occupational Therapy* 2013; 67: 154-161. DOI: 10.5014/ajot.2013.006197.
38. Sygna K, Johansen S and Ruland CM. Recruitment challenges in clinical research including cancer patients and their caregivers. A randomized controlled trial study and lessons learned. *Trials* 2015; 16: 428. DOI: 10.1186/s13063-015-0948-y.
39. Cook WL, Schiller C, McAllister MM, et al. Feasibility of a follow-up hip fracture clinic. *Journal of the American Geriatrics Society* 2015; 63: 598-599. DOI: 10.1111/jgs.13285.
40. Dalhousie University. Wheelchair Specifications Form, http://www.wheelchairskillsprogram.ca (2012, accessed August 1, 2013).
41. World Health Organization. *International classification of functioning disability and health*. Geneva: World Health Organization, 2001.
42. Talo C, Mannarini T and Rochira A. Sense of community and community participation: A meta-analytic review. *Social Indicators Research* 2014; 117: 1-28. DOI: 10.1007/s11205-013-0347-2.
43. Kenny S and Gowran RJ. Outcome Measures for Wheelchair and Seating Provision: A Critical Appraisal. *British Journal of Occupational Therapy* 2014; 77: 67-77. DOI: 10.4276/030802214x13916969447119.
44. Mortenson WB, Miller WC and Auger C. Issues for the selection of wheelchair-specific activity and participation outcome measures: a review. *Archives of Physical Medicine and Rehabilitation* 2008; 89: 1177-1186. DOI: 10.1016/j.apmr.2008.01.010.
45. Miller WC, Garden J and Mortenson WB. Measurement properties of the wheelchair outcome measure in individuals with spinal cord injury. *Spinal Cord* 2011; 49: 995-1000. DOI: 10.1038/sc.2011.45.
46. Auger C, Demers L, Gelinas I, et al. Reliability and validity of the telephone administration of the wheelchair outcome measure (WhOM) for middle-aged and older users of power mobility devices. *Journal of Rehabilitation Medicine* 2010; 42: 574-581. 2010/06/16. DOI: 10.2340/16501977-0557.

47. Fliess-Douer O, Vanlandewijck YC, Lubel Manor G, et al. A systematic review of wheelchair skills tests for manual wheelchair users with a spinal cord injury: towards a standardized outcome measure. *Clinical Rehabilitation* 2010; 24: 867-886. DOI: 10.1177/0269215510367981.
48. Kilkens OJ, Post MW, Dallmeijer AJ, et al. Wheelchair skills tests: a systematic review. *Clinical Rehabilitation* 2003; 17: 418-430. 2003/06/06.
49. Kirby RL, Dupuis DJ, Macphee AH, et al. The wheelchair skills test (version 2.4): measurement properties. *Archives of Physical Medicine and Rehabilitation* 2004; 85: 794-804.

50. Rushton PW, Kirby RL and Miller WC. Manual wheelchair skills: objective testing versus subjective questionnaire. *Archives of Physical Medicine and Rehabilitation* 2012; 93: 2313-2318. DOI: 10.1016/j.apmr.2012.06.007.
51. Rushton PW, Miller WC, Kirby RL, et al. Development and content validation of the Wheelchair Use Confidence Scale: a mixed-methods study. *Disability and Rehabilitation: Assistive Technology* 2011; 6: 57-66. DOI: 10.3109/17483107.2010.512970.
52. Sakakibara BM, Miller WC, Eng JJ, et al. Preliminary examination of the relation between participation and confidence in older manual wheelchair users. *Archives of Physical Medicine and Rehabilitation* 2013; 94: 791-794. DOI: 10.1016/j.apmr.2012.09.016.
53. Berardi A, de Santis R, Tofani M, et al. The Wheelchair Use Confidence Scale: Italian translation, adaptation, and validation of the short form. *Disability and Rehabilitation: Assistive Technology* 2017: 1-6. DOI: 10.1080/17483197.2017.1357053.
54. Jones CA, Feeny D and Eng K. Test-retest reliability of the Health Utilities Index scores: evidence from hip fracture. *International Journal of Technology Assessment in Health Care* 2005; 21: 393-398.

55a. Brazier JE, Harper NM, Jones A, Thomas T, Usherwood T, and Westlake L. 2016. National Centre for Interprofessional Practice and Education. Assessment and Evaluation. The Short-Form (SF-36) Health Survey. https://nexusipe.org/advancing/assessment-evaluation/short-form-sf-36-health-survey.

55b. Brazier, J.E., Harper, N.M., Jones, A., O'Cathain, K.J., Thomas, T., Usherwood, T. & Westlake, L. 1992. Validating the SF-36 health questionnaire: A new outcome measure for primary care. *BMJ*, 305:160.

55c. Katherine Froehlich-Grobe, Elena M Andresen, Charlene Caburnay, Glen W White. Measuring health-related quality of life for persons with mobility impairments: an enabled version of the short-form 36 (SF-36E). Qual Life Res. 2008 Jun; 17(5):751-70. doi: 10.1007/s11136-008-9342-5.

56. Luo N, Johnson J and Coons S. Using instrument-defined health state transitions to estimate minimally important differences for four preference-based health-related quality of life instruments. *Medical Care* 2010; 48: 365-371.
57. Horsman J, Furlong W, Feeny D, et al. The Health Utilities Index: concepts, measurement properties and applications. *Health and Quality of Life Outcomes* 2003; 1: 1-13.
58. Sakakibara BM, Routhier F and Miller WC. Wheeled mobility correlates of life-space and social participation in adult manual wheelchair users aged 50 and older. *Disability and Rehabilitation: Assistive Technology* 2017; 12: 592-598. DOI: 10.1080/17483107.2016.1198434.
59. Sonenblum SE, Sprigle S, Caspall J, et al. Validation of an accelerometer-based method to measure the use of manual wheelchairs. *Med Eng Phys* 2012; 34: 781-786. 2012/06/16. DOI: 10.1016/j.medengphy.2012.05.009.
60. Sonenblum SE, Sprigle S and Lopez RA. Manual wheelchair use: bouts of mobility in everyday life. *Rehabilitation Research and Practice* 2012; 2012: 753165. DOI: 10.1155/2012/753165.
61. Best KL, Routhier F and Miller WC. Older adults' perceptions and experiences with a peer-led wheelchair training program. *34th International Seating Symposium*. Vancouver, BC, 2018.

62. Mortenson WB, Miller WC and Miller-Pogar J. Measuring wheelchair intervention outcomes: Development of the Wheelchair Outcome Measure. *Disability and Rehabilitation: Assistive Technology* 2009; 2: 275-285. DOI: 10.1080/17483100701475863.
63. Giesbrecht EM, Miller WC, Mitchell IM, et al. Development of a wheelchair skills home program for older adults using a participatory action design approach. *Biomed Research International* 2014; 2014: 172434. DOI: 10.1155/2014/172434.
64. Bowman J, Mogensen L, Marsland E, et al. The development, content validity and inter-rater reliability of the SMART-Goal Evaluation Method: a standardised method for evaluating clinical goals. *Australian Occupational Therapy Journal* 2015; 62: 420-427. DOI: 10.1111/1440-1630.12218.
65. Cress ME, Buchner DM, Prohaska T, et al. Best practices for physical activity programs and behavior counseling in older adult populations. *Journal of Aging and Physical Activity* 2005; 13: 61-74.
66. Tabachnick BG and Fidell LS. *Using multivariate statistics*. 5th ed. Boston: Pearson Education, 2007.
67. Vickers AJ. Analysis of variance is easily misapplied in the analysis of randomized trials: a critique and discussion of alternative statistical approaches. *Psychosomatic medicine* 2005; 67: 652-655. 2005/07/28. DOI: 10.1097/01.psy.0000172624.52957.a8.
68. Mbuagbaw L, Aves T, Shea B, et al. Considerations and guidance in designing equity-relevant clinical trials. *International Journal for Equity in Health* 2017; 16: 93. 2017/06/07. DOI: 10.1186/s12939-017-0591-1.
69. Government of Canada. Infection prevention and control for COVID-19: Interim guidance for outpatient and ambulatory care settings. Canada.ca. 2020-05-23. https://www.canada.ca/en/public-health/services/diseases/2019-novel-coronavirus-infection/guidance-documents/interim-guidance-outpatient-ambulatory-care-settings.html
70. Government of Canada. Coronavirus disease (COVID-19): For health professionals. Canada.ca. 2020-06-10. https://www.canada.ca/en/public-health/services/diseases/2019-novel-coronavirus-infection/health-professionals.html
71. Government of Canada. Coronavirus disease (COVID-19): For health professionals. Canada.ca. 2020-06-10. https://www.canada.ca/en/public-health/services/diseases/2019-novel-coronavirus-infection/guidance-documents/interim-guidance-outpatient-ambulatory-care-settings.html
72. Public health ethics framework: A guide for use in response to the COVID-19 pandemic in Canada. 2020-06-05. https://www.canada.ca/en/public-health/services/diseases/2019-novel-coronavirus-infection/canadas-reponse/ethics-framework-guide-use-response-covid-19-pandemic.html

Auger, C., Demers, L., Gelinas, I., Routhier, F., Mortenson, W. B., & Miller, W. C. (2010). Reliability and validity of the telephone administration of the wheelchair outcome measure (WhOM) for middle-aged and older users of power mobility devices. *Journal of Rehabilitation Medicine*, *42*(6), 574-581. https://doi.org/10.2340/16501977-0557

Best, K. L., Miller, W. C., Routhier, F., & Eng, J. J. (2017). Feasibility of the trial procedures for a randomized controlled trial of a community-based peer-led wheelchair training program for older adults. *Pilot and Feasibility Studies*, *4*, 18. https://doi.org/10.1186/s40814-017-0158-3

Giesbrecht, E. M., & Miller, W. C. (2017). A randomized control trial feasibility evaluation of an *m*Health intervention for wheelchair skill training among middle-aged and older adults. *PeerJ*, *Oct 5*(5), e3879. https://doi.org/10.7717/peerj.3879

Giesbrecht, E. M., Miller, W. C., Mitchell, I. M., & Woodgate, R. L. (2014). Development of a wheelchair skills home program for older adults using a participatory action design approach. *Biomed Research International*, *2014*, 172434. https://doi.org/10.1155/2014/172434

Kirby, R. L., Dupuis, D. J., Macphee, A. H., Coolen, A. L., Smith, C., Best, K. L., Newton, A. M., Mountain, A. D., Macleod, D. A., & Bonaparte, J. P. (2004). The wheelchair skills test (version 2.4): measurement properties. *Archives of Physical Medicine and Rehabilitation*, *85*(5), 794-804. <Go to ISI>://MEDLINE:15129405

Lemay, V., Routhier, F., Noreau, L., Phang, S. H., & Ginis, K. A. (2012). Relationships between wheelchair skills, wheelchair mobility and level of injury in individuals with spinal cord injury. *Spinal Cord*, *50*(1), 37-41. https://doi.org/10.1038/sc.2011.98

Sakakibara, B. M., Routhier, F., & Miller, W. C. (2017). Wheeled mobility correlates of life-space and social participation in adult manual wheelchair users aged 50 and older. *Disability and Rehabilitation: Assistive Technology*, *12*(6), 592-598. https://doi.org/10.1080/17483107.2016.1198434

73. World Health Organization. Disability considerations during the COVID-19 outbreak. www.who.int; https://www.who.int/docs/default-source/documents/disability/covid-19-disability-briefing.pdf.
74. Government of Canada. Hard-surface disinfectants. https://www.canada.ca/en/health-canada/services/drugs-health-products/disinfectants/covid-19.html#a3
75. Samsung: Keep your Galaxy devices clean. https://www.samsung.com/us/support/answer/ANS00086342/
